# Inhibition Effect of Graphene on Space Charge Injection and Accumulation in Low-Density Polyethylene

**DOI:** 10.3390/nano8110956

**Published:** 2018-11-20

**Authors:** Zhonglei Li, Jingang Su, Boxue Du, Zhaohao Hou, Chenlei Han

**Affiliations:** Key Laboratory of Smart Grid of Education Ministry, School of Electrical and Information Engineering, Tianjin University, Tianjin 300072, China; houzhaohao@163.com (Z.H.); chenleiyn@163.com (C.H.)

**Keywords:** low-density polyethylene, graphene, nanocomposites, space charge, charge mobility, interface region

## Abstract

Space charge injection and accumulation is attracting much attention in the field of dielectric insulation especially for electronic devices, power equipment and so on. This paper proposes using the inhibition effect of graphene for the injection and accumulation of space charge in low-density polyethylene (LDPE). Scanning electron microscope (SEM) and transmission electron microscopy (TEM) images were employed to observe the dispersion of graphene with a two-dimensional structure in LDPE. The time-dependent space charge dynamic behaviors of graphene/LDPE nanocomposites with the filler content of 0, 0.003, 0.005, 0.007 and 0.01 wt % were characterized by the pulsed electro-acoustic (PEA) test at 40, 60 and 80 °C, and the charge mobility was evaluated by its depolarization processes. The experimental results show that for the undoped LDPE film, large amounts of space charges were injected from the electrodes into samples, especially at 60 and 80 °C. The graphene/LDPE nanocomposites with a filler content of 0.005 wt % could markedly suppress the space charge injection and accumulation even at 80 °C, which is attributed to the large quantities of graphene-polymer in interface regions. These interface regions introduced numbers of deep trap sites within the forbidden band of nanocomposites, which can reduce the de-trapping rate of charges and suppress the space charge accumulation in the polymer bulks. The graphene/LDPE nanocomposites are suggested for dielectric applications, intending the inhibition of space charge injection and accumulation.

## 1. Introduction

Polyethylene-based insulating materials, such as low-density polyethylene (LDPE), high-density polyethylene (HDPE), cross-linked polyethylene (XLPE) and so on, are widely used for electrical insulation, due to their excellent electrical and mechanical properties [1,2,3]. Among the electrical properties, space charge characteristics are attracting much attention along with the rapid development of direct-current electronic devices and power equipment during the past decade. Space charge is considered as the excess electric charge with a continuum of charge distribution over a region (either a volume or an area) of dielectric insulation, which will distort the electric field distribution and accelerate the aging, degradation and even breakdown of the insulation [4,5]. The space charge generally comes from the charge injection from electrodes and the ionization of chemical groups under a DC electric field of up to tens of kilovolts per millimeter [6,7,8]. With an increase of the voltage level, the miniaturization of electrical devices and the rise of operation temperatures, the space charge injection and accumulation will be further exacerbated and become one of the critical issues for the development of PE-based insulation. Therefore, the suppression of space charge injection and accumulation has been a research focus in the field of electrical insulation.

Nanodielectric insulation has been one of the research hotspots to develop the advanced polymeric insulation, especially for the PE-based insulation of high-voltage direct-current (HVDC) extruded cable [9,10,11]. As early as 2005, Y. Ohki et al. found that LDPE and XLPE modified by inorganic oxide nanoparticles has a much higher breakdown strength, lower conductivity, and less space charge accumulation than undoped polymer [12,13]. The inorganic nanoparticles, including magnesium oxide (MgO), silica (SiO_2_), zinc oxide (ZnO) etc., are considered to be optimal additives for HVDC cable insulation [12,13,14,15,16]. The excellent electrical properties of nanodielectrics were ascribed to the large quantities of polymer-nanofiller interface regions owning to the high specific surface area of nanofillers. Based on the multi-core model proposed by T. Tanaka, the polymer-nanofiller interface regions can bring in numbers of deep traps, which capture the free charge carriers and enhance the partial discharge resistance of polymers [17]. However, a large amount of nanoparticle filling, even with surface treatment, may lead to non-uniform dispersion or local agglomeration, which would weaken the effects of nanofiller-polymer interface regions or even play an adverse effect on electrical properties [14,17]. The relatively new class of two-dimensional nanofillers is gradually attracting attention in the field of nanodielectrics.

Graphene, with a unique two-dimensional structure and a giant specific surface area, allows for theoretically higher interactions with a polymer matrix than the traditional nanofillers [18,19,20]. It is therefore speculated that a the addition of a tiny amount of graphene (much lower than the amount of traditional nanoparticles) could bring a large quantity of nanofiller-polymer interface regions, but also avoid the agglomeration of nanofillers, thus exploiting further potentials of nanodielectrics. Recent investigations have proved that graphene with a content of 0.005 wt % could increase the deep trap level and reduce the carrier mobility of LDPE nanocomposites [21,22]. However, local agglomeration occurs in 0.01 wt % graphene/LDPE nanocomposites, resulting in a decrease of the deep trap level [21]. There is still a limit to the understanding of correlation between the space charge characteristics and its trap level distribution in graphene/LDPE nanocomposites, which is important for the extruded insulation, especially at the operating temperature.

This paper proposes the inhibition effect of graphene for the injection and accumulation of space charge in LDPE. The space charge behaviors of graphene/LDPE nanocomposites with different filler content of 0, 0.003, 0.005, 0.007 and 0.01 wt % were investigated by the pulsed electro-acoustic (PEA) method at 40, 60 and 80 °C. Additionally, the trap-controlled carrier mobility of LDPE nanocomposites was evaluated by the depolarization processes of space charge.

## 2. Materials and Methods

### 2.1. Sample Preparation of Graphene/LDPE Nanocomposites

In this study, the LDPE powders with density of 0.92 g/cm^3^ were provided by Dow Chemical Company (DFDB-6005 NT, Midland, MI, USA). The graphene synthesized by using a modified hummers approach were purchased from Tanfeng Graphene Technology Co., LTD. (Nanjing, China), of which the dimensions are 0.5–2 nm in thickness and 0.5–5 μm in diameter. The specific surface area of graphene is 1000~1217 m^2^/g.

The surface treatment of graphene was carried out using sodium lauryl-sulfonate (SLS, C_12_H_25_SO_3_Na) as a kind of anionic surfactant. The graphene was dissolved in ethanol by sonication for 30 min to disperse. And the SLS/ethanol mixture was slowly added into a graphene/ethanol mixture, which was put into a water bath in advance at about 75 °C, then magnetically stirred for 12 h and sonicated for 1 h. The mass ratio of the SLS to the graphene nanofillers is 1:100. The surface-modified graphene was dried in a vacuum oven at 80 °C for 12 h to remove solvent. Then the treated graphene was mixed with the LDPE powder with different nanofiller contents of 0.003, 0.005, 0.007 and 0.01 wt %. Then the melt blending was conducted in a twin-screw continuous mixer with a rotation speed of 40 rpm at 160 °C for 20 min. The mixed compounds were hot-pressed in a stainless-steel mold with a thickness of 250 μm at 160 °C under 15 MPa for 20 min. The cool down process was done with pressure for 1 h.

### 2.2. Characteristics

The microstructure of the cross-section of graphene/LDPE nanocomposites was observed by high-resolution scanning electron microscopy (SEM, GeminiSEM 500, ZEISS, Oberkochen, Germany). The freezing microtome section of nanocomposites is investigated by using a Transmission electron microscopy (TEM, Tecnai F20 S/TEM, FEI, Columbia, MD, USA) to represent the arrangement of the graphene within the matrix.

Thermal analysis of the films was performed with a Differential Scanning Calorimeter (DSC, DSC 12E, Mettler, Schwerzenbach, Switzerland). Temperature ranged from 20 °C up to 200 °C and vice versa for cooling scans performed with a heating/cooling rate of 10 °C/min in a nitrogen atmosphere. A three minute-time with temperature remaining at 200 °C was applied to erase the history of the thermal behavior of the samples (7 mg) for evaluating changes during the cooling scans. Endotherms were represented with upward curves in the scans.

**Figure 1:**
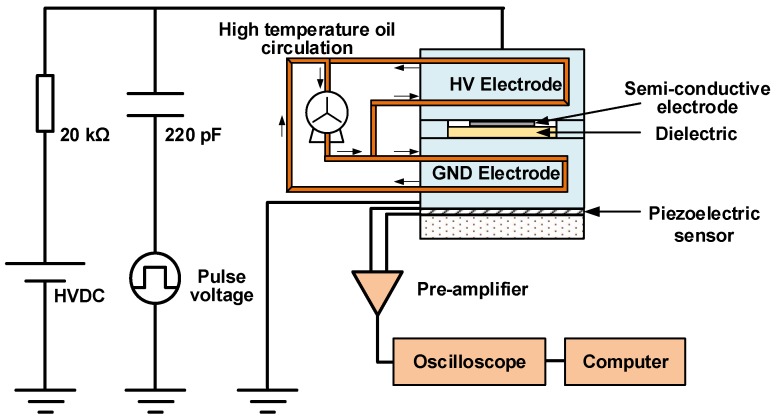
Schematic illustration of the experimental setup for PEA.

The space charge characteristics of graphene/LDPE nanocomposites at 40, 60 and 80 °C were measured by the PEA method proposed by T. Takada [23]. The experimental setup is presented in Figure 1. The HVDC power source is used to form a DC electric field and inject space charges into the samples. Then the impulses were applied to the samples to excite the space charges and generate the acoustic wave with the space charge information, which propagated through the dielectric to the piezoelectric sensor. The output signal of the sensor were pre-amplified and finally converted to the space charge distribution in the dielectrics. The anode (HV electrode) was made of semiconductive material, and the cathode (GND electrode) was made of aluminum. The voltage-on test of each sample was performed under 30 kV/mm for 30 min, and the short-circuit test continued for 15 min. An oil circulation system with a constant temperature oil-bathing unit was employed to control the experimental temperature.

### 2.3. Charge Mobility Evaluation by Space Charge Dissipation

The carrier mobility of graphene/LDPE nanocomposites can be calculated according to the space charge dissipation process, approximately [24,25]. The carrier mobility is expressed by the following equation [24,25]:

(1) $$\mu (t)=\frac{2\varepsilon }{q(t)q^{\prime }(t)}\cdot \;\frac{dq(t)}{dt}\;$$

where *ε* is the permittivity of sample. *q*’(*t*) = *q*^+^(*t*) − *q*^−^(*t*), where *q*^+^(*t*) and *q*^−^(*t*) are the average charge densities of positive and negative charges. *q*(*t*) stands for the average charge density in the specimen at a depolarization time of *t*, which is calculated by:

(2) $$q(t)=\frac{1}{L}\int_{0}^{L}\left|q\left(x,t\right)\right|dx\;$$

where *L* is the thickness of samples, and the thermal expansion at experimental temperatures is neglected. *q*(*x*, *t*) is the space charge density in the specimen where *x* stands for location and *t* stands for depolarization time.

The Equation (1) should be under the assumptions that [24]:

(a) The carrier mobility *μ* is considered as constant in space and varies only with time.
(b) The volume density of trapped charges *ρ* is much larger than that of free charges *ρ_f_*.
(c) The hole-electron recombination is ignored. Hence, the charge dissipation only comes from the bulk transport of space charges to the ground. The total current density *J_T_(t)* is equal to *L·dq(t)*/*dt*.

Furthermore, we made a simplification, i.e., *q*’(*t*) = *q*(*t*) [24]. This assumption is valid when unipolar charges are dominated in the polymer bulks. Then:

(3) $$\mu (t)=\frac{2\varepsilon }{q{\left(t\right)}^{2}}\cdot \frac{dq(t)}{dt}\;$$

Additionally, the trap level involving the depolarization process can be obtained by [25]:

(4) $$\varphi (t)=kT\ln\left(\frac{\mu (t)}{veR^{2}}kT\right)\;$$

where *k* is the Boltzmann constant. *T* is the absolute temperature. *ν* is the escape frequency of trapped charges, which is equal to *kT*/*h*. *h* is the Planck constant. *e* is the electronic charge quantity. *R* is the average distance of localized states. Even though some of the above assumptions are very strong and do not reflect the real conditions in most cases, the Equations (3) and (4) still could be used to evaluate the trap-controlled carrier mobility in polymer approximately.

## 3. Results

### 3.1. Characterization of Graphene/LDPE Nanocomposites

**Figure 2:**
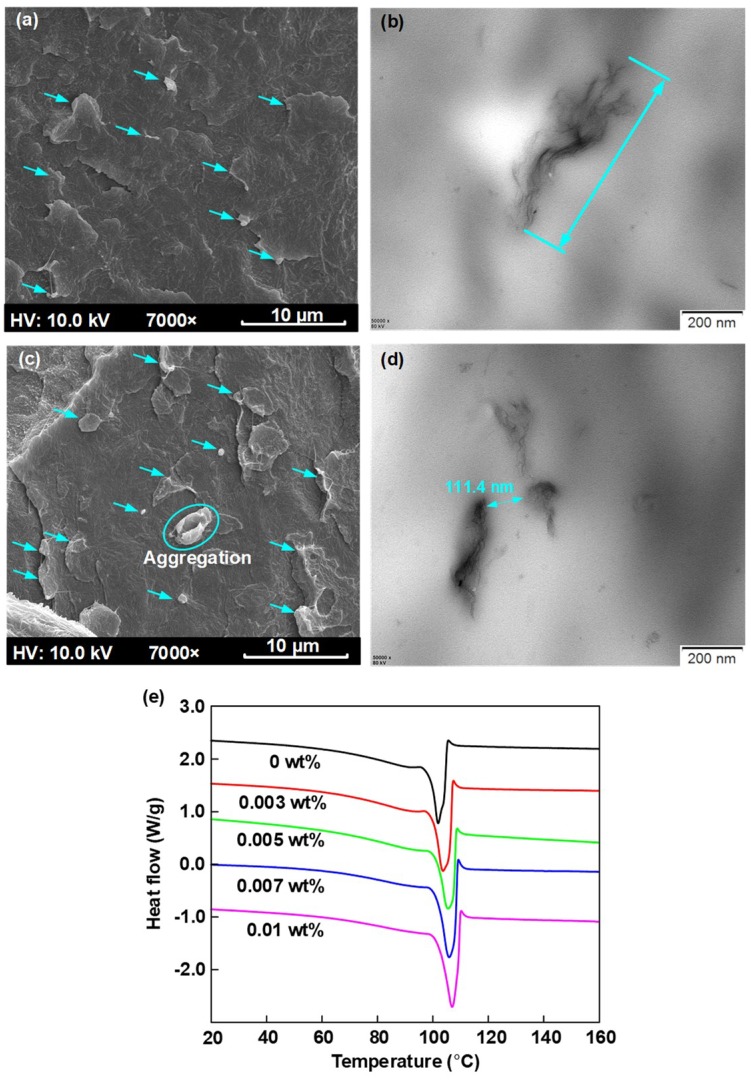
(**a**,**b**) SEM and TEM images of the cross section of the 0.005 wt % graphene/LDPE Nanocomposites. (**c**,**d**) SEM and TEM images of the freezing microtome section of the 0.01 wt % graphene/LDPE Nanocomposites (**e**) DSC curves of Nanocomposites as a function of graphene content.

Figure 2a–d shows the SEM and TEM images of the cross section of the 0.005 and 0.01 wt % graphene/LDPE nanocomposites. It was observed that the graphene fillers marked by arrows were well-dispersed in the 0.005 wt % nanocomposites. The TEM image shown in Figure 2b presents the 2-D structure of graphene nanofillers. As the graphene content increased to 0.01 wt %, the graphene fillers presented in SEM image (Figure 2c) increased, and the distance between the neighboring fillers decreased as shown in Figure 2d.

Figure 2e presents the DSC curves of graphene/LDPE nanocomposites. It is found that the melting peak temperature increased by 5.3 °C with the filler content increasing from 0 to 0.01 wt %. Through the integral of DSC curves, the crystallinity (*X*) can be calculated by the formula as follows [26]:

(5) $$X=\frac{\Delta H_{m}}{\Delta H_{0}}\times 100\%\;$$

where ∆*H*_0_ is the melting enthalpy of LDPE fully crystallized (100%) and generally ∆*H*_0_ = 293 J/g [27]. ∆*H_m_* is the melting enthalpy of LDPE nanocomposites investigated. The degree of crystallinity characterized by DSC tests was 36.5%, 37.0%, 37.2%, 41.2% and 42.5%, for 0 wt %, 0.003 wt %, 0.005 wt %, 0.007 wt % and 0.01 wt % for graphene/LDPE Nanocomposites, respectively. The incorporation of graphene increased the crystallinity of PE nanocomposites. It is indicated that the well-dispersed graphene fillers acted as the heterogeneous nucleating agents, thus accelerating the rate of crystallization and enhancing the crystallinity of LDPE composites.

### 3.2. Space Charge Accumulation in Graphene/LDPE Nanocomposites

**Figure 3:**
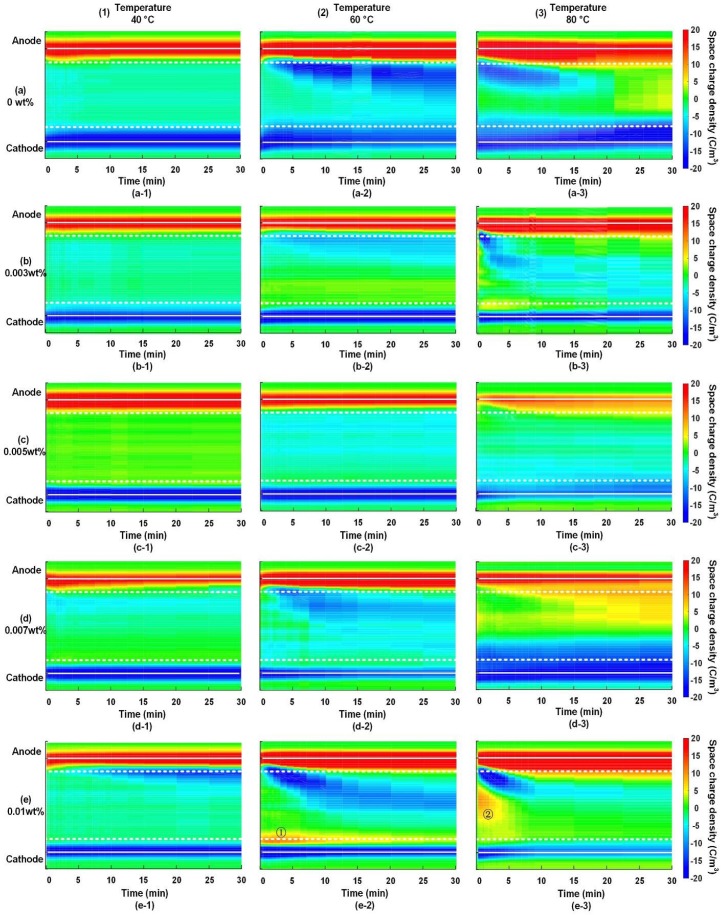
Space charge dynamic behaviors in graphene/LDPE composites with different filler content under +30 kV/mm at 40, 60 and 80 °C, respectively. (**a-1**–**a-3**) 0 wt %, (**b-1**–**b-3**) 0.003 wt %, (**c-1**–**c-3**) 0.005 wt %, (**d-1**–**d-3**) 0.007 wt %, and (**e-1**–**e-3**) 0.01 wt % graphene/LDPE nanocomposites.

The space charge dynamic behaviors of graphene/LDPE nanocomposites with different filler content at various temperatures are shown in Figure 3. The color map with a defined color bar were employed to present both the space charge polarity and amount in nanocomposites. The vertical axis of each drawing stands for the thickness dimension of specimens, and the horizontal axis is for the time dimension. The white dashed lines show the position in the vicinity of electrode, of which the space charge amount varies with polarization time can be used to characterize the charge injection and transportation from both electrodes to bulk.

It is obvious that both the ambient temperature and graphene addition had a marked effect on the injection and accumulation of space charge. For the 0, 0.003, 0.005 and 0.007 wt % samples at temperature of 40 °C shown in Figure 3(a-1)–(d-1), most space charges were accumulated in vicinity of both electrodes and few space charges were injected into the bulk. Therefore, both the polarity and the amount of space charge remained stable under applied voltage. It is indicated that the space charge injection and transportation was inactive under 30 kV/mm at 40 °C. With the rise of the ambient temperature from 40 to 80 °C, an obvious aggravation of space charge injection and accumulation occurred in the neat LDPE. As shown in Figure 3(a-2)–(a-3), both the polarity and the amount of space charge vary with the polarization time.

The space charge behaviors at 60 and 80 °C were prominently modified by the introduction of 0.003 and 0.005 wt % graphene, as shown in Figure 3b,c. However, 0.007 and 0.01 wt % graphene additions played a negative role in modifying the space charge characteristics. The effects of the ambient temperature and graphene on the space charge dynamic behaviors in graphene/LDPE nanocomposites were further analyzed as following.

#### 3.2.1. Effect of Temperature on Space Charge Accumulation

**Figure 4:**
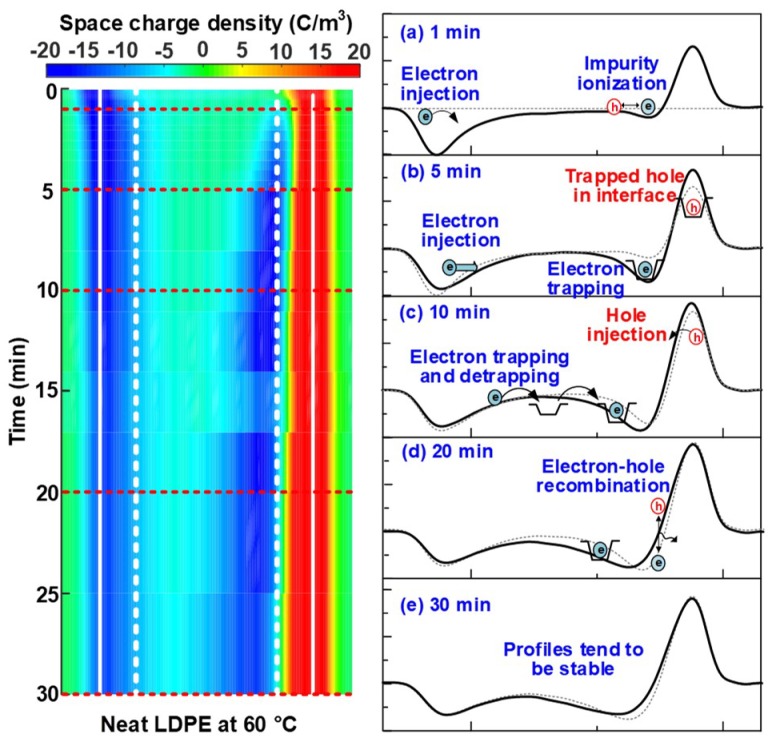
Space charge dynamic behaviors in the neat LDPE sample under +30 kV/mm at 60 °C. The polarization time is (**a**) 1 min, (**b**) 5 min, (**c**) 10 min, (**d**) 20 min and (**e**) 30 min, respectively.

**Figure 5:**
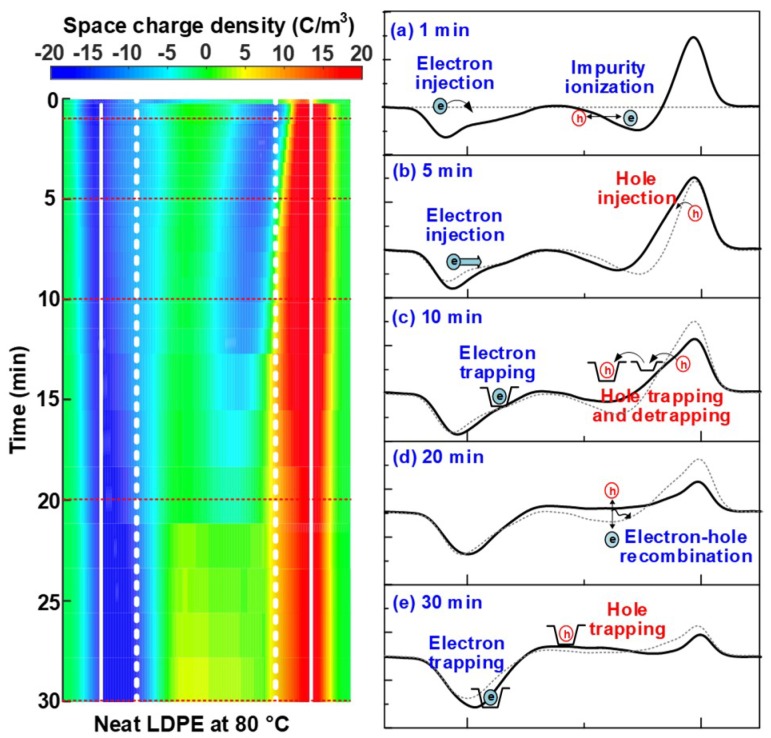
Space charge dynamic behaviors in the neat LDPE sample under +30 kV/mm at 80 °C. The polarization time is (**a**) 1 min, (**b**) 5 min, (**c**) 10 min, (**d**) 20 min and (**e**) 30 min, respectively.

For the neat LDPE, few space charges were injected into the bulk at 40 °C, but an aggravated injection and accumulation of space charge occurs at 60 and 80 °C, as shown in Figure 3(a-2)–(a-3). In order to further investigate the mechanism of ambient temperature on space charge characteristics, the space charge dynamic behaviors in the neat LDPE sample at 60 and 80 °C were analyzed and compared as following. The space charge profiles of neat LDPE at the polarization time of 1, 5, 10, 20 and 30 min are illustrated in Figure 4a–e and Figure 5a–e respectively, in which the dashed lines stand for the space charge profiles at the previous polarization time.

For the neat LDPE at 60 °C shown in Figure 4, an evident injection of electrons from the cathode occurred at a polarization time of 1 min. Meanwhile, heterocharges were observed at the vicinity of the anode, which was considered to be resulting from the space charge injection and impurity ionization. With the lapse of polarization time, numbers of electrons injected from cathode were transported towards anode and trapped, giving rise to the accumulation of heterocharges at the side of the anode. Besides, negative charges generated by impurity ionization were trapped and accumulated near the anode. As a result, the electric field nearby anode was enhanced severely, which would reduce the potential barrier for Schottky injection and further lead to an injection of holes from the anode after the electric field was applied for 10 min, as shown in Figure 4b,c. Consequently, recombination processes between electrons and holes occurred at some recombination sites in the vicinity of anode, and homocharges become dominate at the position nearby the anode marked with the white dashed line, as shown in Figure 4. The profiles of space charge tended to be relatively stable up to 30 min. This phenomenon was exactly similar to the results reported by [8], in which the space charge polarity in the vicinity of anode reversed with the temperature rising from 30 to 50 °C for XLPE under 30 kV/mm. The results indicated that the rise of ambient temperature from 40 to 60 °C had a significant effect on the charge injection and accumulation in neat LDPE samples, especially for electrons.

With the increase of temperature to 80 °C, the injection and transportation of electrons (including both injection and ionization) were further accelerated, thus leading to more severe space charge accumulation at the polarization time of 1 min. What is even more remarkable was that the large quantities of positive charges injected from the anode into samples, as shown in Figure 5b. As a result, the heterocharges, at the position nearby anode marked with the white dashed line, were replaced by homocharges after the stress was applied for 10 min, as shown in Figure 5c. With the lapse of polarization time, a large quantity of positive charges transported further towards the middle of LDPE sample, and an obvious electron-hole recombination processes occurred within the polymer bulks. As a result, positive charges became the dominant status after the electric field was applied for 30 min. The results shown above indicate that the rising of temperature from 60 to 80 °C played more significant roles in accelerating the injection and transportation of holes than that of electrons in neat LDPE samples.

#### 3.2.2. Effect of Graphene on Space Charge Accumulation

For the graphene/LDPE composites with different filler content at 40 °C, most homocharges were accumulated near the electrodes, and few space charges were injected into the bulks except for the 0.01 wt % graphene/LDPE nanocomposites shown in Figure 3(e-1).

At temperature of 60 °C, negative charges became dominated due to the acceleration of ionization and electron injection. By comparing the space charge color images in Figure 3(a-2)–(c-2), we could observe the profound effect of graphene addition on space charge injection and transportation. For the 0.005 wt % graphene/LDPE nanocomposites, the charge amount at the location marked with the white dashed line remained unchanged with the time, and the small amount of negative charges were considered to come from impurity ionization, as shown in Figure 3(c-2). It is suggested that the incorporation of graphene with a tiny filler content can effectively suppresses the transportation of space charges (including both injection and ionization), which was attributed to the deep trap sites introduced by the large quantities of polymer-graphene interface regions. With a further increase of graphene from 0.005 to 0.01 wt %, the accumulated space charge amount within samples increased, indicating an aggravation of space charge injection and transportation. On the one hand, the agglomeration between neighboring fillers and the local overlapping between the interface regions would weaken the effect of the deep trap sites. On the other hand, the agglomerated graphene provided percolating paths for charge carriers in a local region, thus aggravating the space charge transportation.

The rising of temperature from 60 to 80 °C would further accelerate the injection and transportation of holes. It can be observed in Figure 3(a-3)–(c-3) that, with the increase of filler content to 0.005 wt %, the injection depth of space charges reduced significantly, especially for holes. For the neat LDPE, heterocharges accumulated nearby the anode side in the initial stage, which resulted in a severe distortion of the local field. With the injection of large quantities of holes from the anode, homocharges became dominant at the location near the anode, thus leading to a marked decrease of electric field at the anode side and an increase at the cathode side. The space charge injection and local field distortion are considered to be the key factors involving the insulating properties of dielectrics under DC field. It can be observed in Figure 3(c-3) that homocharges were dominant in the area near both electrodes and that the electric field kept constant, approximately in 0.005 wt % nanocomposites. The graphene with a content of 0.005 wt % could effectively resist the charge injection and transportation from the anode into the polymer.

As the filler content reached to 0.01 wt %, homocharge injection and transportation were significantly accelerated under the co-effect of electric field and high temperature, as shown in Figure 3(d-3). In this case, the electric field in the middle of specimen was be greatly enhanced. For the 0.01 wt % nanocomposites, one interesting phenomenon to note is that, positive charges appeared at the cathode side at the initial stage of polarization processes and moved towards the cathode under the stress of electrostatic force (see ① and ② in Figure 3e). It is probably due to the strong ionic dissociation of the surfactant of sodium lauryl-sulfonate at high temperature.

The results shown above indicate that the 0.005 wt % graphene/LDPE nanocomposites had the fewest amount of injected space charges and the weakest electric field distortion at any ambient temperature, due to the effect of the interaction zones between graphene and LDPE matrix. However, as the filler content reaches 0.01 wt %, it may lead to an acceleration of the homocharge injection and transportation, thus aggravating the space charge injection and accumulation in LDPE nanocomposites.

### 3.3. Effect of Graphene on the Carrier Mobility

**Figure 6:**
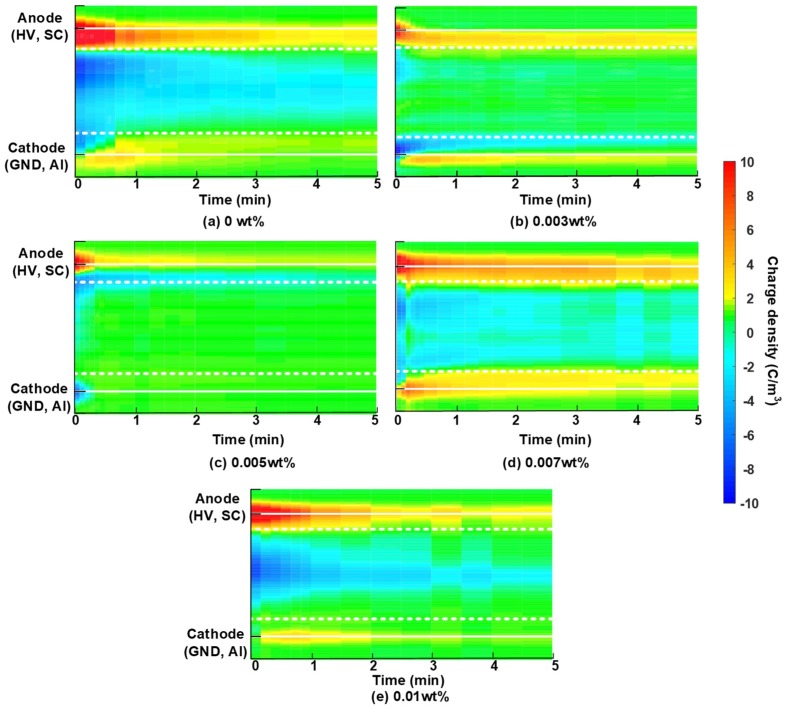
Space charge dissipation in LDPE composites with the different filler content at 60 °C. (**a**) 0 wt %, (**b**) 0.003 wt %, (**c**) 0.005 wt %, (**d**) 0.007 wt % and (**e**) 0.01 wt % graphene/LDPE nanocomposites.

The space charges dissipation process can provide an estimation of the apparent trap controlled mobility and trap level by using Equations (3) and (4). According to the assumptions explained in Section 2.3, unipolar charges should be dominated in the polymer bulks and charge recombination is neglected. From Figure 2, the accumulated space charges within sample bulks at 60 °C were dominated by negative charges. Therefore, the space charge behaviors at the shorted-circuit condition at 60 °C (shown in Figure 6) was employed to analyze the effect of graphene addition on carrier mobility. It could be observed that during the depolarization process of LDPE nanocomposites, positive charges located at the electrode-dielectrics interfaces, and negative charges accumulated within sample bulks, which is in agreement with the polarization processes shown above. The injection amount and depth of negative space charges decreased significantly with the increase of graphene content from 0 to 0.005 wt %, but increased with the further increase of filler content from 0.005 to 0.01 wt %.

**Figure 7:**
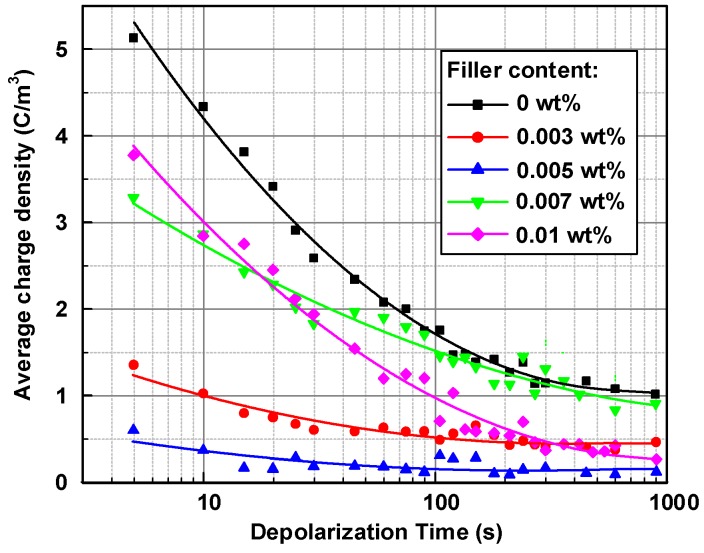
Relationship between the average charge density and depolarization time of graphene/LDPE composites at the shorted-circuit condition at 60 °C.

**Figure 8:**
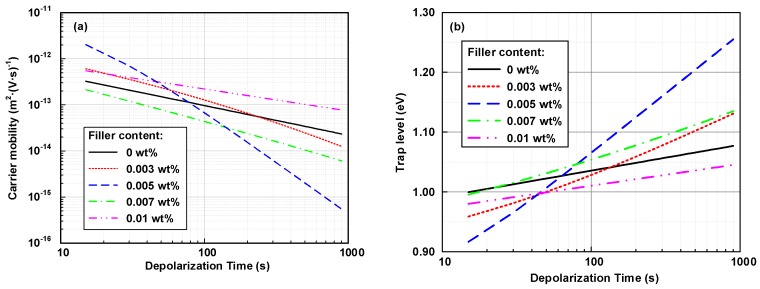
(**a**) Carrier mobility and (**b**) trap level of graphene/LDPE composites with different filler content at the shorted-circuit condition at 60 °C.

The average charge densities (*q*(*t*)) in LDPE samples during the depolarization process of 15 min were calculated by Equation (2) and presented in Figure 7, and the carrier mobility and the corresponding trap energy of graphene/LDPE nanocomposites at 60 °C are shown in Figure 8. It can be observed in Figure 7 that the average charge density within neat LDPE sample exceeded 5 C/m^3^ at 5 s, and decayed with time nonlinearly. The decay rate of charge density declined with time, due to the deeper traps involved in the depolarization process. It was also observed that the 0.003, 0.005 and 0.007 wt % graphene/LDPE nanocomposites all had much lower initial charge densities and slower charge decay rates than that of neat LDPE. As shown in Figure 7, the order of residual charge density up to 15 min of depolarization time is 0 wt % > 0.007 wt % > 0.003 wt % > 0.01 wt % > 0.005 wt %.

Figure 8a presents that the carrier mobility decreased with depolarization time. During the depolarization time varying from 100 to 1000 s, the carrier mobility was in the following order, 0.01 wt % > 0 wt %> 0.003 wt % > 0.007 wt % > 0.005 wt %. In the first few seconds, the 0.003 and 0.005 wt % nanocomposites had relatively high values, probably due to the charge depolarization at the electrode-polymer interfaces. The relationship between the trap depth and the depolarization time shown in Figure 8b shows that the 0.005 wt % graphene/LDPE nanocomposites had a much deeper trap level than the undoped LDPE sample. However, a further increase of the graphene content lead to a decrease of deep trap level. As the content reached 0.01 wt %, the trap-controlled carrier mobility became higher than the undoped sample.

## 4. Discussion

The space charge behaviors of graphene/LDPE nanocomposites indicated that a negative charge was always dominant in the samples under applied voltage at 40 and 60 °C. It is also concluded that the injection and transportation of positive charges (hole) became more active than electron as the ambient temperature rises to 80 °C. It is due to the hole having a high injection barrier at polymer-semiconductive material interface and a lower transfer rate in LDPE bulks than the electrons at lower temperature. With the rise of ambient temperature, the de-trapping and transfer rate of holes (including both injection and ionization) were significantly enhanced, resulting in a significant injection and transportation of holes at 80 °C. With the hole injection from the electrode into samples, the hole-electron recombination occurred in the vicinity of the anode, thus leading to the replacement of heterocharges with homocharges in the nearby anode.

**Figure 9:**
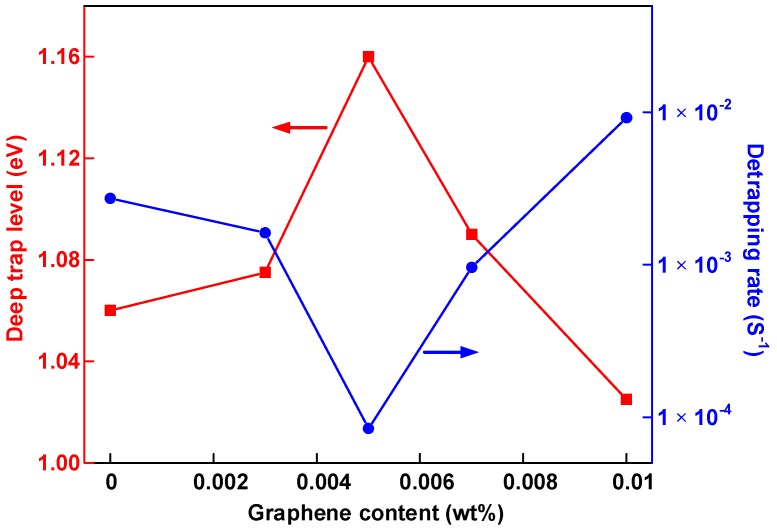
The deep trap level of graphene/LDPE nanocomposites as well as the de-trapping rate (*P_de_*) at 60 °C.

The addition of only a tiny amount graphene made a profound improvement of space charge characteristics, which was attributed to the improved trap levels and decreased carrier mobility. According to the multi-core model proposed by T. Tanaka, the micro interface regions between the graphene nanofillers and polymer matrix, with a thickness of tens of nanometers, contains a large number of deep charge traps. The deep traps introduced by the well-dispersed fillers interact with the intrinsic trapping sites in LDPE matrix and result in a dramatic increase of deep trap density in composites, as shown in Figure 7. The de-trapping rate (*P_de_*) of charges from traps can be expressed as [28]:

(6) $$P_{de}=\nu_{ATE}e^{-E_{T}/kT}\;$$

where *E_T_* is the energy level of traps, *ν_ATE_* is the attempt-to escape frequency, which can be represented as [28]:

(7) $$\nu_{ATE}={\left(kT\right)}^{3}/\left(dh^{3}v^{2}\right)\;$$

where *d* is the number of defect movement directions. *h* is the Planck’s constant. *ν* is the vibration frequency around the defects at the orthogonal plane flaw of moving direction. The *ν_ATE_* can be considered as a constant that is equal to 2.85 × 10^13^ s^−1^ at 60 °C (333.15 K) [28]. The trap depth of each sample at 300 s in Figure 8b was set as the deep trap level. The deep trap level of graphene/LDPE nanocomposites as well as the de-trapping rate (*P_de_*) at 60 °C are presented in Figure 9. It is indicated that, with the increase of the filler content from 0 to 0.005 wt %, the deep trap level increased from 1.06 to 1.16 eV, following with the decrease of the de-trapping rate from 2.7 × 10^−3^ to 8.4 × 10^−5^ s^−1^. The lower the de-trapping rate in dielectrics, the more difficult the charge injection and transport. With a further increase of graphene from 0.005 to 0.01 wt %, the local overlapping between the interface regions or even the agglomeration (shown in Figure 2) lead to a decrease of deep trap level and a higher de-trapping rate, resulting in an aggravation of space charge injection and transportation.

The experimental results and discussions both indicate that the surface-treated graphene with a content of 0.005 wt % could significantly inhibit space charge injection and accumulation in LDPE. As concluded in the references [12,13,14,15,16], for the traditional inorganic nanoparticles, including MgO, SiO_2_, ZnO, the optimum content is in the range of 0.5~5 wt % for the modification of space charge behaviors. Owing to the advantage of two-dimensional structure and an enormous specific surface area shown in Figure 2b, graphene with a content of only 0.005 wt % could introduce a large number of microscopic interfaces between graphene fillers and LDPE polymer, which has great potential applications for the DC electronic devices and power equipment insulation.

## 5. Conclusions

In this paper, graphene/LDPE nanocomposites were prepared. The space charge dynamic behaviors of graphene/LDPE nanocomposites at ambient temperature of 40, 60 and 80 °C were investigated. The effects of graphene content on the space charge characteristics of LDPE were analyzed, and an inhibition effect of graphene with a content of 0.005 wt % on the injection and accumulation of space charge in LDPE is proposed. The main results and conclusions can be summarized as follows:

1. Negative charge was always dominant in the samples under voltage applied at 40 and 60 °C, while the hole injection and transportation became more active than electron as the temperature rises to 80 °C, resulting in the replacement of heterocharges with homocharges in the vicinity of the anode.
2. The graphene filling with a filler content of 0.005 wt % brought in a large quantity of filler-polymer interface regions containing deep trap sites, which could reduce the detrapping rate of charges and effectively inhibited the space charge injection and accumulation in the polymer.
3. Large amounts of heterocharges were formed in 0.01 wt % graphene/LDPE nanocomposites under 30 kV/mm at 60 and 80 °C, and the trap-controlled carrier mobility became higher than the undoped sample.
